# *Pyrus ussuriensis* Maxim 70% ethanol eluted fraction ameliorates inflammation and oxidative stress in LPS‐induced inflammation in vitro and in vivo

**DOI:** 10.1002/fsn3.3077

**Published:** 2022-09-23

**Authors:** Fei Peng, Xin Ren, Bin Du, Yuedong Yang

**Affiliations:** ^1^ Hebei Key Laboratory of Active Components and Functions in Natural Products Hebei Normal University of Science and Technology Qinhuangdao China; ^2^ Collaborative Innovation Centre of Hebei Chestnut Industry Hebei Normal University of Science and Technology Qinhuangdao China

**Keywords:** acute lung injury, anti‐inflammatory, MAPKs, Nrf2/HO‐1, *Pyrus ussuriensis* Maxim

## Abstract

*Pyrus ussuriensis* Maxim (PUM) is a popular fruit among consumers, and also used as medical diet for dissolving phlegm and arresting cough. The present study aims to investigate the potential protective effect of *P. ussuriensis* Maxim 70% ethanol eluted fraction (PUM70) on lipopolysaccharide (LPS)‐induced alveolar macrophages and acute lung injury (ALI) in mice. A total of 18 polyphenol compounds were tentatively identified in PUM70 by mass spectrometry (MS) analysis. The results in vivo suggested that PUM70 treatment could effectively alleviate the histological changes, and significantly inhibit the activity of myeloperoxidase (MPO) and the expression of pro‐inflammatory cytokines (tumor necrosis factor‐α (TNF‐α), interleukin‐1β (IL‐1β), and interleukin‐6 (IL‐6)). The cell test results show that PUM70 exerted its protective effect by suppressing the messenger RNA (mRNA) expression levels (inducible nitric oxide synthase (iNOS) and cyclooxygenase‐2 (COX‐2) and decreasing nitric oxide (NO) and prostaglandin 2 (PGE2) contents. In addition, it also inhibited the overproduction of pro‐inflammatory cytokines (TNF‐α, IL‐1β, and IL‐6). Furthermore, PUM70 induced the production of heme oxygenase 1 (HO‐1) protein and nuclear translocation of Nrf2 (nuclear factor erythroid 2‐related factor 2), indicating that PUM70 could mitigate oxidative injury via the Nrf2/HO‐1 pathway. Moreover, PUM70 inhibited LPS‐induced inflammation by blocking the phosphorylation of mitogen‐activated protein kinases (MAPKs). The above results indicate that PUM70 has protective effects on LPS‐induced ALI, possibly be related to the inhibition of MAPK and Nrf2/HO‐1 signaling pathways.

## Graphical abstract

In this work, we demonstrated that PUM70 specifically suppressed LPS‐mediated inflammatory responses. Notably, PUM70 treatment inhibited LPS‐induced lung damage, pro‐inflammatory cytokine production, and reduced macrophage infiltration in the lungs. Mechanistically, PUM70 suppressed LPS‐induced inflammation and oxidative stress via the Nrf2/HO‐1 and MAPKs pathway. Our results provide solid evidence that PUM70 is a potential preventive or curative natural substance for ALI.

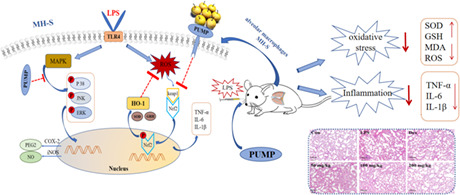

## 1 INTRODUCTION

Acute lung injury (ALI) has been a major problem in the field of intensive care medicine, which is characterized by pulmonary edema, respiratory distress, and progressive hypoxemia (Burnham et al., 2014; Thille et al., 2013). Although the treatment strategy for ALI is being continuously improved, there are still some side effects during medication such as drug resistance, metabolic disorder, and osteoporosis, resulting in an extremely high mortality rate. Therefore, ALI has always been a hot and challenging issue in medical research, and it is imperative to develop safe, effective, and new therapeutic drugs.

Acute lung injury (ALI) is induced by various etiologies, among which pneumonia and sepsis caused by bacterial infection are the most common pathogenic factors (Rubenfeld et al., 2005). Lipopolysaccharide (LPS) is a potent inducer of ALI, which can cause strong inflammation in the body. Currently, establishment of airway inflammation through LPS stimulation has been widely applied to the creation of ALI animal models.

The release of a large number of inflammatory cytokines, such as interleukin‐1β (IL‐1β), tumor necrosis factor‐α (TNF‐α), and so on, has been proved to be one of the key factors promoting the development of ALI (Lei et al., 2018). In recent years, the development of ALI drugs has been mainly targeted at the control of degree of inflammation (Wu et al., 2019). Numerous studies have been reported that oxidative stress is one of the main causes of inflammatory response, which could cause lesions to DNA, proteins, and lipids by regulating antioxidant enzyme production (Kwon et al., 2018; Yang et al., 2016). Therefore, some antioxidant pathways potentially mediated by endogenous or exogenous compounds may be utilized as the therapeutic targets to address ALI (Kim et al., 2020).

Naturally active ingredients such as flavonoids and polyphenols have strong anti‐inflammatory effects with high safety and effectiveness, and can simultaneously act on multiple targets in the inflammatory pathway (Li et al., 2016; Qian et al., 2019). *Pyrus ussuriensis* Maxim (Anli in Chinese) is an important nutritional and economic fruit crop in China, which has been used as medical diet for dissolving phlegm and arresting cough. In our previous study, the extract of *P. ussuriensis* Maxim was found to contain high concentrations of both total phenolics and pentacyclic triterpenoids (Peng et al., 2020). However, in spite of the promising results observed in the anti‐inflammatory and antioxidant activity of this pear, researches in this field are still scarce. Therefore, to be included in the market for these uses, more studies focused on their specific effects in vitro and in vivo are needed. In this work, we investigated the potential protective effect of *P. ussuriensis* Maxim extract against LPS‐induced alveolar macrophages and ALI in mice.

## 2 MATERIALS AND METHODS

### 2.1 Reagents

Compounds such as MTT (3‐[4,5‐dimethylthiazol‐2‐yl]‐2,5 diphenyl tetrazolium bromide), LPS, Griess reagent, and Evans blue (≥95%) were purchased from Sigma (St. Louis, MO, USA), The assay kits used for the detection of reactive oxygen species (ROS), superoxide dismutase (SOD), malondialdehyde (MDA), myeloperoxidase (MPO), and glutathione (GSH) were obtained from Nanjing Jiancheng Bioengineering Institute (Nanjing, China). The enzyme‐linked immunosorbent assay (ELISA) kits for nitric oxide (NO), prostaglandin 2 (PGE2), TNF‐α, IL‐1β, and interleukin‐6 (IL‐6) were obtained from Solarbio Science & Technology Co., Ltd. (Beijing, China). The antibodies were obtained from Santa Cruz Biotechnology, Inc. (Santa Cruz, CA, USA). CelLytic™ NuCLEAR™ Extraction Kit was purchased from Sigma‐Aldrich Co., LLC. (Beijing, China). Pierce Bicinchoninic Acid (BCA) Protein Assay kit was purchased from Servicebio Technology Co., Ltd. (Wuhan, China).

### 2.2 Collection of plant materials and preparation of extracts

The *P. ussuriensis* Maxim (PUM) fruits were collected on their commercial maturity in September 2019, from Qinhuangdao City, Hebei Province, China, and kept at 4°C before use. The extraction was conducted according to the methods of our previous study (Peng et al., 2018). In brief, the ethyl acetate fraction was further purified using microporous resin with an ethanol–water gradient (0, 100 to 90: 10, v/v). The 70% ethanol eluted fraction was collected and freeze‐dried (Alpha 2‐4 Dplus, Christ, Germany) to obtain PUM70.

### 2.3 Analysis of PUM70

The identification of components in PUM70 was performed using Ultra Performance LC system (Thermo Fisher U3000, USA) and Exactive™ mass spectrometer equipped (Thermo Fisher Scientific, USA). The data were subsequently input into the Mass Frontier 7.0 software and combined with ChemSpider network database. The total ion current chromatograms for PUM70 are shown in Figure S1.

### 2.4 Animals

Prior to the experiment, experimental use of animals was approved by the Ethics Committee of Hebei Normal University of Science and Technology. Male BALB/c mice (20–24 g) were purchased from Vitong Lihua Laboratory Animal Technology Co., Ltd (Beijing, China). The mice were kept in a clean animal house and fed with sterile food and water. During the test period, the temperature and relative humidity of the environment were kept at 20–24°C and 40%–70%, respectively, with a light‐and‐darkness cycle of 12 h daily. After a period of acclimatization, the mice were randomly divided into control group, LPS group, dexamethasone (5 mg/kg) group, and PUM70 (50, 100, and 200 mg/kg) groups, eight for each group. Equal volumes of normal saline (control group and LPS group), dexamethasone, and PUM70 were administered intragastrically once a day. On the seventh day, the mice were anesthetized and fixed, then LPS (10 μg LPS was dissolved in 50 μl phosphate‐buffered saline (PBS)) was injected intratracheally through a microsprayer needle (model IA‐1C, Penn‐Century, USA) attached to a microsyringe (FMJ‐250, Penn‐Century, USA) to establish the experimental groups, or injected with sterile water to serve as the control group. After the induction of injury for 6 h, the mice were sacrificed. The blood and lung tissues were collected and stored at −80°C.

### 2.5 Histopathological observation

The upper lobe tissues of the right lung were soaked in 4% formaldehyde solution and fixed for 48 h, then dehydrated with ethanol, made transparent with xylene, dipped in wax, embedded, sliced (5 μm), retrieved, and dried. Sections were stained with hematoxylin–eosin (HE) and observed under an inverted microscope.

### 2.6 Inflammatory index in lung

The bronchial tubes were flushed with 2 ml cold PBS buffer three times to collect the bronchoalveolar lavage fluid (BALF). One part of BALF was taken to test the protein content with the BCA protein assay kit, and the other part of BALF was taken to measure the pro‐inflammatory cytokine content according to the ELISA kit instruction. The neutrophil count was determined on a smear prepared by Wright–Giemsa stain.

### 2.7 Oxidative stress index in the lung

The left lung tissue was taken and rinsed with PBS and then ground with an abrasive rod. The cell suspension was filtered with 300 μm Nilon net and added with fluorescent probe dichlorodihydrofluorescein diacetate (DCFH‐DA). The cell precipitate was collected after centrifugation and washed with PBS twice to be used for flow cytometry. The results were shown as the average fluorescence intensity value (the fluorescence intensity in each cell). The lower lobe of the right lung was ground with liquid nitrogen to prepare the tissue homogenate. MDA, SOD, GSH, and MPO were detected, respectively, according to the reagent instructions.

### 2.8 Cell culture and cell viability

The mouse alveolar macrophages (MH‐S) were obtained from the Procell Life Science & Technology Co., Ltd. (Wuhan, China). Subsequently, cell culture and cell viability tests were determined following the reported method (Kwon et al., 2018). Briefly, the MH‐S cells (1 × 10^5^ cells per well) were seeded into a 96‐well plate. After 24 h of incubation at 37°C, 10 μl of PUM70 at various concentrations was added to each well of the plates. After incubation for additional 24 h, the PUM70‐treated cells were incubated with 100 μl MTT (0.5 mg/ml) for 4 h. After the removal of supernatant, MTT–formazan was dissolved in 200 μl dimethyl sulfoxide (DMSO). A microplate reader (ELX‐800, BioTek, Vermont, USA) was used to measure the absorbance at 544 nm (Figure S2).

### 2.9 Oxidative stress index in cells

The cells were lysed with RIPA (radioimmunoprecipitation assay) lysis buffer and then the supernatant was collected after centrifugation for the detection of oxidative stress index. MH‐S cells were treated with PUM70 (2.5, 5, and 10 μg/ml) for 2 h and stimulated with 2 μg/ml LPS for 1 h. Then, the cells were incubated with 10 M DCFH‐DA for 30 min at 37°C. After centrifugation, the precipitates were collected and washed twice with PBS. The intracellular ROS content was analyzed by flow cytometry (CytoFLEX LX; Beckman Coulter, USA) and spectrofluorometer.

### 2.10 Inflammatory index in cells

MH‐S cells (1 × 10^5^ cells/well) were pretreated with PUM70 (in gradient concentrations) for 2 h, treated with 2 μg/ml of LPS, and incubated for 24 h. After centrifugation, the supernatant was collected. The cell lysis solution was mixed with Griess reagent and incubated for 15 min. The absorbance was measured at 540 nm by the Epoch microplate reader (PerkinElmer, USA), and the level of NO was calculated according to the standard curve. For the PEG2 assay, cells were pretreated with PUM70 (in gradient concentrations) for 2 h, treated with 2 μg/ml of LPS, and incubated for 24 h. After centrifugation, the supernatant was collected. The levels of PGE2 and pro‐inflammatory cytokines were measured with the ELISA kit.

### 2.11 qRT‐PCR

The quantitative reverse transcription‐polymerase chain reaction (qRT‐PCR) assay was performed according to the reported method (Yang et al., 2016). Relative fold‐changes were calculated according to 2^−ΔΔCT^.

### 2.12 Western blotting

MH‐S cells were prepared in petri dishes 12 h prior to the assay and treated with PUM70 for 24 h, followed by stimulation with LPS (2 μg/ml) for 1 h. The western blotting was performed in accordance with Kwon et al. (2018). Cytoplasmic proteins and nuclear proteins were fractionated using a CelLytic™ NuCLEAR™ Extraction Kit. Pierce BCA Protein Assay Kit was used to determine the protein concentration. The extracted samples were fractionated by 10% sodium dodecyl sulfate/polyacrylamide gel electrophoresis (SDS/PAGE) and transferred to Immunoblot polyvinylidene difluoride (PVDF) films. After blocking with 5% (w/v) skim milk dissolved in Tris‐buffered saline containing Tween 20 (TBS‐T) for 1 h, immunoblotting was performed using appropriate primary antibodies (1:2000). The membranes were incubated overnight (4°C), washed with TBS‐T, and immersed in horseradish peroxidase (HRP)‐conjugated secondary antibody (1:2500, 1 h). Antibody‐binding proteins were visualized by ECL Advanced Western blotting detection reagent (Advanta, CA, USA). The density of the immunoreactive bands was analyzed using ImageJ software (NIH, Bethesda, MD, USA).

### 2.13 Statistical analysis

The SPSS 20.0 version (SPSS Inc., Chicago, IL, USA) was used to analyze the Pearson correlation coefficients. The significant differences data were performed for comparative analysis using Duncan's multiple range test. Statistically, *p* < .05 indicated a significant difference.

## 3 RESULTS

### 3.1 Identification of components in PUM70

As a result in Table 1, eighteen compounds were identified in the PUM70, including three flavan‐3‐ols (A‐ and B‐type procyanidin dimer, catechin), six phenolic acids (quinic acid, 1‐caffeoylquinic acid, 3,5‐di‐*O*‐caffeoylquinic acid, ferulic acid, feruloylquinic acid, and sinapic acid hexoside), one anthocyanin (cyanidin 3‐*O*‐glucoside), and eight flavonol derivatives (kaempferol derivatives, quercetin derivatives, and isorhamnetin derivatives).

**TABLE 1:** Identification of compounds in *Pyrus ussuriensis* Maxim 70% ethanol eluted fraction (PUM70)

No.	*t*_R_ (min)	[*M* − *H*]^−^ (m/z)	Proposed compound	MS fragment	Tentative identification
1	1.20	191.0183	C_7_H_12_O_6_	173.0106, 127.9617	Quinic acid
2	1.74	515.1379	C_25_H_24_O_12_	353.0863, 191.0552	3,5‐di‐*O*‐Caffeoylquinic acid
3	2.64	353.0884	C_16_H_18_O_9_	191.0551, 179.0343	1‐Caffeoylquinic acid
4	4.28	193.0464	C_10_H_10_O_4_	–	Ferulic acid
5	4.43	423.0922	C_15_H_14_O_6_	–	(+)‐Catechin
6	5.24	289.0679	C_17_H_22_O_10_	223.0607	Sinapic acid hexoside
7	5.59	577.1338	C_30_H_26_O_12_	407.0757, 289.0731	B‐type procyanidin dimer
8	6.14	449.1071	C_21_H_22_O_11_	287.057169	Cyanidin 3‐*O*‐glucoside
9	6.50	863.1923	C_45_H_36_O_18_	577.1374, 289.0721	A‐type procyanidin trimer
10	6.89	609.1342	C_27_H_30_O_16_	301.0362	Quercetin 3‐*O*‐rutinoside
11	7.30	623.1586	C_28_H_32_O_16_	315.0431	Isorhamnetin 3‐*O*‐rutinoside
12	7.89	477.1034	C_22_H_22_O_12_	315.0476	Isorhamnetin 3‐*O*‐galactoside
13	8.26	447.0908	C_21_H_20_O_11_	285.0394	Kaempferol 3‐*O*‐glucoside
14	8.46	505.0965	C_23_H_23_O_13_	463.0843, 301.0354	Quercetin‐acylated‐hexoside
15	10.24	463.0852	C_21_H_20_O_12_	301.0355	Quercetin 3‐*O*‐glucoside
16	11.98	367.1336	C_17_H_20_O_9_	191.0541, 174.9556	Feruloylquinic acid
17	12.86	489.1071	C_23_H_22_O_12_	285.0394	Kaempferol‐3‐*O*‐6‐ acetylglucoside
18	13.23	519.1141	C_24_H_24_O_13_	315.0571	Isorhamnetin‐acylated‐hexoside

*Note*: [M − H]^−^ indicates identification in negative ionization modes.

### 3.2 Pathological effect of PUM70 in ALI mice

**FIGURE 1:**
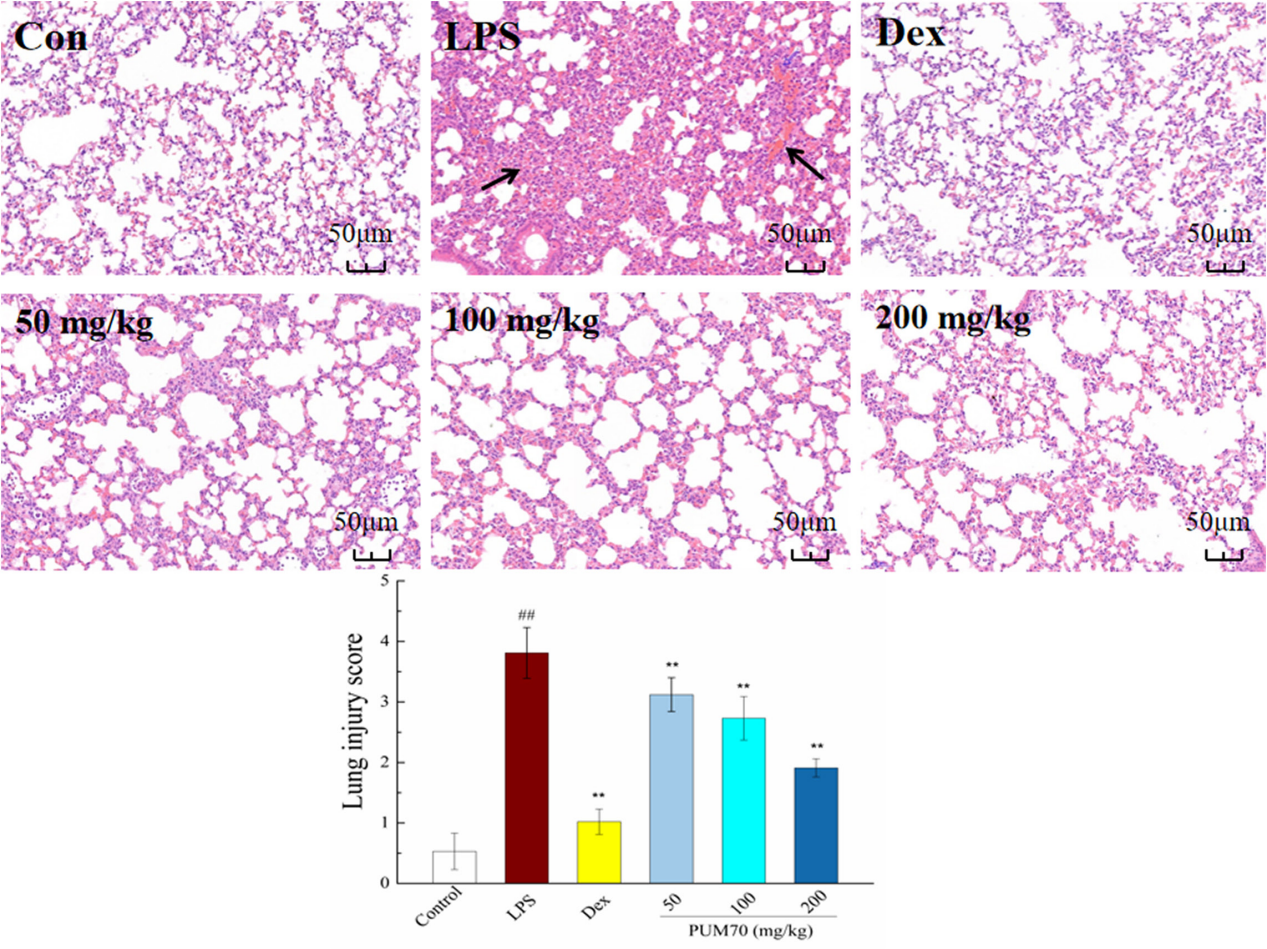
Pathological changes of acute lung injury in mice. Data are from one representative experiment of three replicates (*n* = 3 mice for each group)

As shown in Figure 1, there was no significant pathological change in the lung tissue of the normal control group. However, significant pathological changes of the lung tissues appeared in the LPS group, such as alveolar volume decrease, alveolar septum thickening, and extensive inflammatory cell infiltration. Interestingly, the pathological changes of mice were greatly alleviated in each PUM70 group, including the decreases in inflammatory cells, bronchoalveolar wall thickness, and pulmonary congestion (Figure 1). With increasing PUM70 concentration, the inhibitory effect tended to become more significant.

### 3.3 Effects of PUM70 on inflammatory index in the lung

**FIGURE 2:**
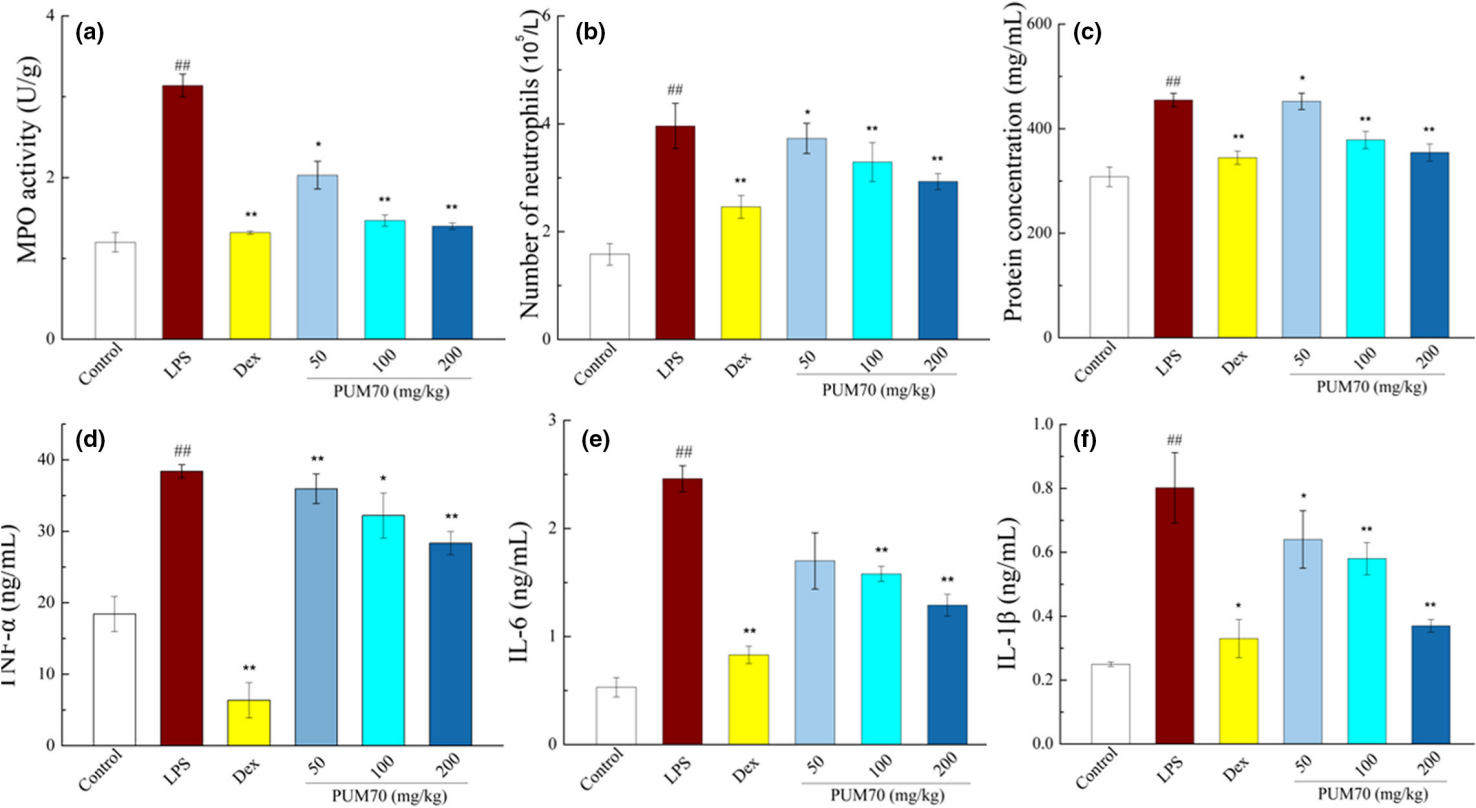
Protein content, neutrophil number, and inflammatory factors in bronchoalveolar lavage fluid (BALF) and myeloperoxidase (MPO) activity. (a) MPO activity; (b) number of neutrophils; (c) protein concentration; (d) contents of tumor necrosis factor‐α (TNF‐α); (e) contents of interleukin‐6 (IL‐6); and (f) contents of interleukin‐1β (IL‐1β). Data are provided from 5 mice per group and presented as mean ± SD. ^##^ *p* < .01 versus the control group; **p < .01, *p < .05 versus the lipopolysaccharide (LPS)‐treated group

It can be seen from Figure 2a, there was a significant upregulation in MPO activity (*p* < .01), while PUM70 could significantly inhibit MPO activity (*p* < .05). Moreover, the LPS group had significantly more neutrophils and higher protein concentration in the BALF than the control group, while PUM70 exhibited a dose‐dependent inhibitory effect on the neutrophil number and protein concentration relative to the LPS group (Figure 2b,c). The ELISA results (Figure 2d–f) revealed that LPS induced the expression of TNF‐α, IL‐6, and IL‐1β in the BALF (*p* < .01); on the contrary, PUM70 pretreatment significantly downregulated their expression, and the alleviating effect was correlated with the dose of PUM70.

### 3.4 Effects of PUM70 on oxidative stress index in the lung

**FIGURE 3:**
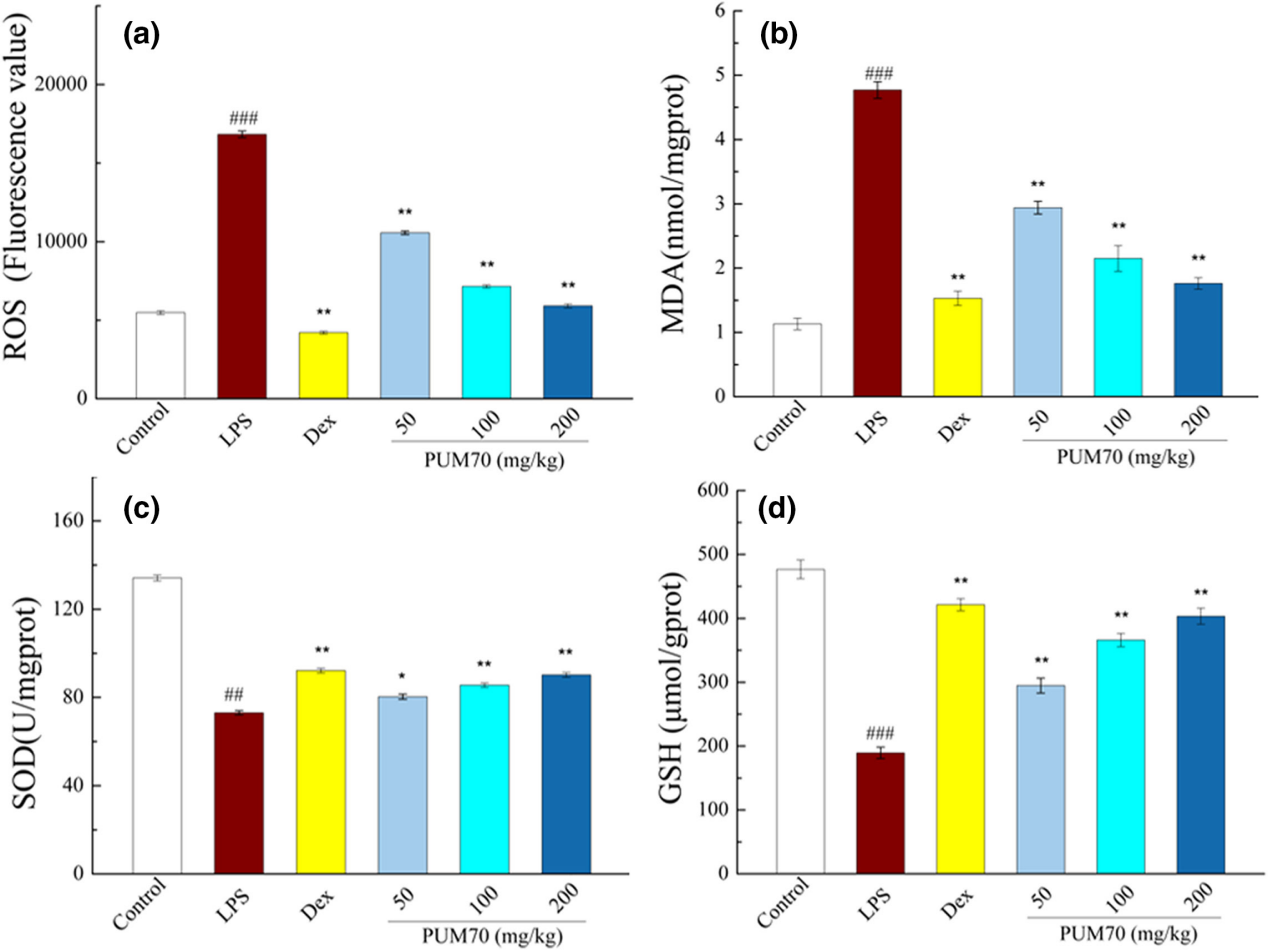
Effect of *Pyrus ussuriensis* Maxim 70% ethanol eluted fraction (PUM70) on the oxidative stress index in lung. (a) The average fluorescence intensity of reactive oxygen species (ROS) level; (b) malondialdehyde (MDA) content; (c) superoxide dismutase (SOD) activity; and (d) glutathione (GSH) content. Data are provided from 5 mice per group and presented as mean ± SD. ^###^ *p* < .001, ^##^ *p* < .01 compared with the control group; **p < .01, *p < .05 compared with the lipopolysaccharide (LPS)‐treated group

The flow cytometry results showed that the average fluorescence value of ROS was significantly increased in the LPS treatment group (*p* < .001), while pretreatment with PUM70 could effectively reduce ROS (*p* < .01) (Figure 3a). Similarly, the LPS group had significantly decreased SOD activity and GSH content, while increasing MDA content than the control group. However, the situation had significantly improved after PUM70 treatment (Figure 3b–d).

### 3.5 Effect of PUM70 on the oxidation index of LPS‐induced alveolar macrophages

**FIGURE 4:**
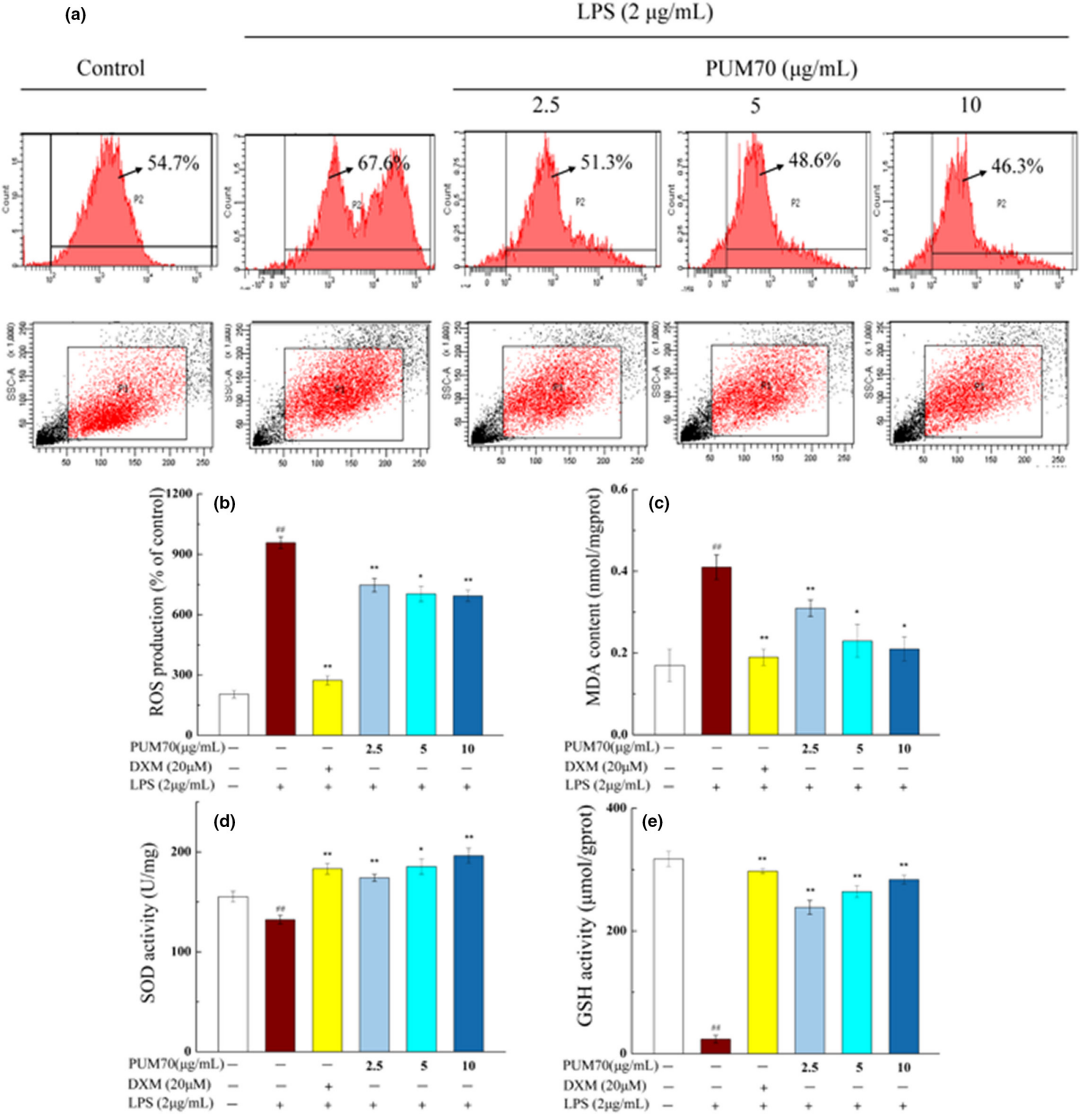
Effect of *Pyrus ussuriensis* Maxim 70% ethanol eluted fraction (PUM70) on the oxidative stress index in lipopolysaccharide (LPS)‐induced alveolar macrophages. Cells were treated with PUM70 (50, 100, and 200 μg/ml) for 2 h and stimulated with 2 μg/ml LPS for 24 h. Data are presented as mean ± SD. (a) The plot shows the histogram of gated cells; (b) the production of reactive oxygen species (ROS); (c) the levels of malondialdehyde (MDA); (d) the activity of superoxide dismutase (SOD); (e) the contents of glutathione (GSH). ^##^ *p* < .01 compared with the control group; **p < .01, *p < .05 compared with the lipopolysaccharide (LPS)‐treated group

The elevation of ROS level caused by changes in different signaling pathways facilitates the formation of a pro‐inflammatory microenvironment and promotes the progress of disease development (Kwon et al., 2018). As can be seen from Figure 4a, the curve of LPS‐treated cells showed a significant shift to the right, indicating a significant increase (by 67.6%) in intracellular ROS level relative to that in the control (by 54.7%). The addition of PUM70 at three doses decreased the ROS level to 51.3% (50 μg/ml), 48.6% (100 μg/ml), and 46.3% (200 μg/ml) compared with the LPS group, respectively (Figure 4b). The concentrations of SOD, MDA, and GSH are key indicators of oxidative stress. We also determined the MDA content, SOD and GSH activity of lipid peroxidation products in the cells. It can be seen from Figure 4c–e that the MDA level was doubled in the cells treated with LPS, while there were significant decreases in SOD and GSH activity (*p* < .01). Interestingly, administration of PUM70 at different concentrations significantly rescued the LPS‐induced alterations. Importantly, the PUM70 treatment could reduce the MDA content at 100 and 200 μg/ml, and significantly increased SOD and GSH activity from 132.38 to 196.35 (U/mg), 23.54 to 283.88 (μmol/gprot), respectively, in a dose‐dependent inhibitory relationship.

### 3.6 Effects of PUM70 on the inflammatory index in cells

**FIGURE 5:**
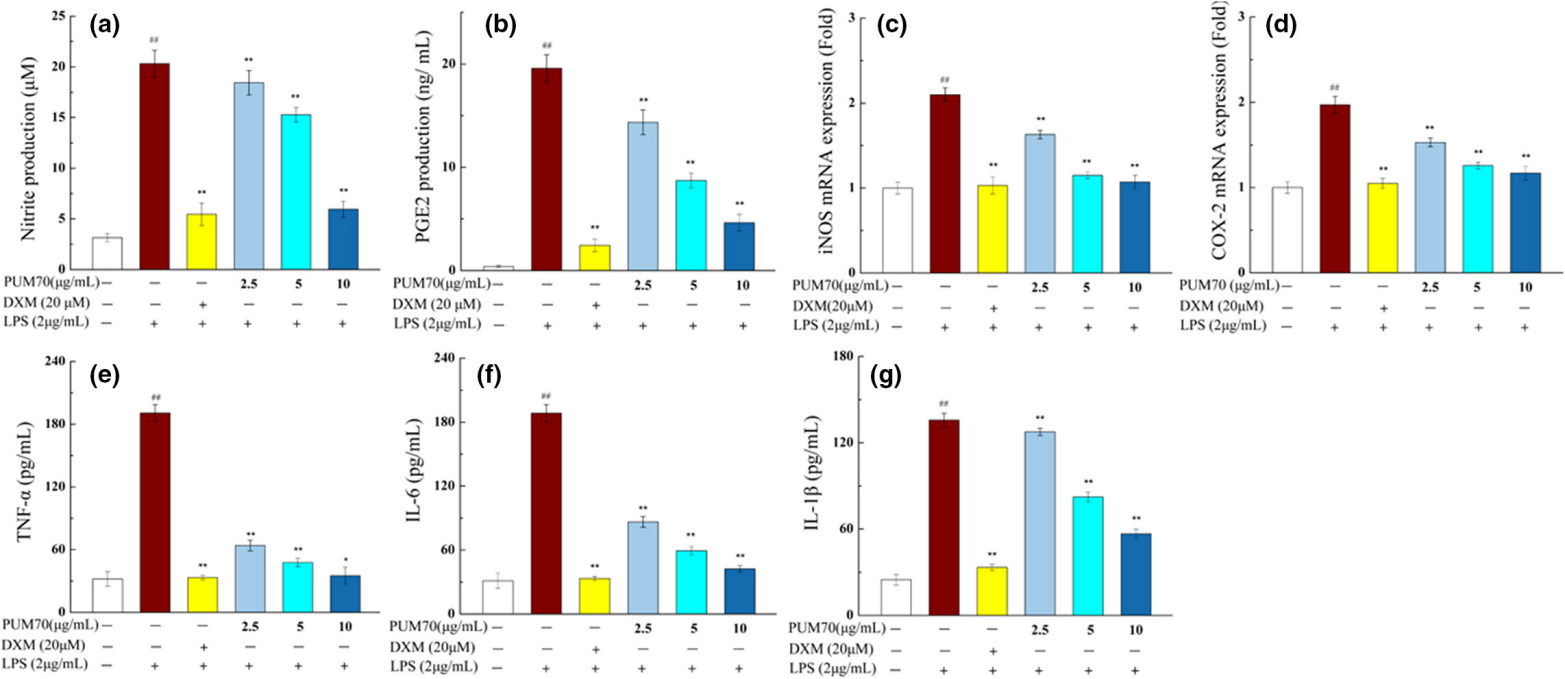
Effect of *Pyrus ussuriensis* Maxim 70% ethanol eluted fraction (PUM70) on inflammatory index in lipopolysaccharide (LPS)‐induced inflammatory mediators. Data are presented as mean ± SD. (a) Nitric oxide (NO) content; (b) prostaglandin 2 (PGE2) content; (c) messenger RNA (mRNA) expression of inducible nitric oxide synthase (iNOS); (d) mRNA expression of cyclooxygenase‐2 (COX‐2); (e‐g) content of tumor necrosis factor‐α (TNF‐α), interleukin‐6 (IL‐6), and interleukin‐1β (IL‐1β). ^##^ *p* < .01 compared with the control group; **p < .01, *p < .05 compared with the LPS‐treated group

Figure 5a,b indicate that the content of NO and PGE2 was significantly increased in the cells treated with LPS (*p* < .01). However, PUM70 treatment could significantly suppress the NO and PGE2 production (*p* < .01). We further determined whether PUM70 could suppress inducible nitric oxide synthase (iNOS) and cyclooxygenase‐2 (COX‐2) protein expression. The qRT‐PCR analysis revealed that pretreatment with PUM70 could significantly (*p* < .01) suppress the upregulation of iNOS and COX‐2 mRNA level (Figure 5c,d), and that the alleviating effect was correlated with the dose of PUM70. The ELISA results suggested that the content of TNF‐α, IL‐6, and IL‐1β in the supernatant of cell culture was significantly increased in the LPS treatment group (*p* < .01). By contrast, administration of PUM70 at different concentrations significantly rescued the LPS‐induced alterations (Figure 5e–g).

### 3.7 Effect of PUM70 on the expression of HO‐1 and Keap1/Nrf2

**FIGURE 6:**
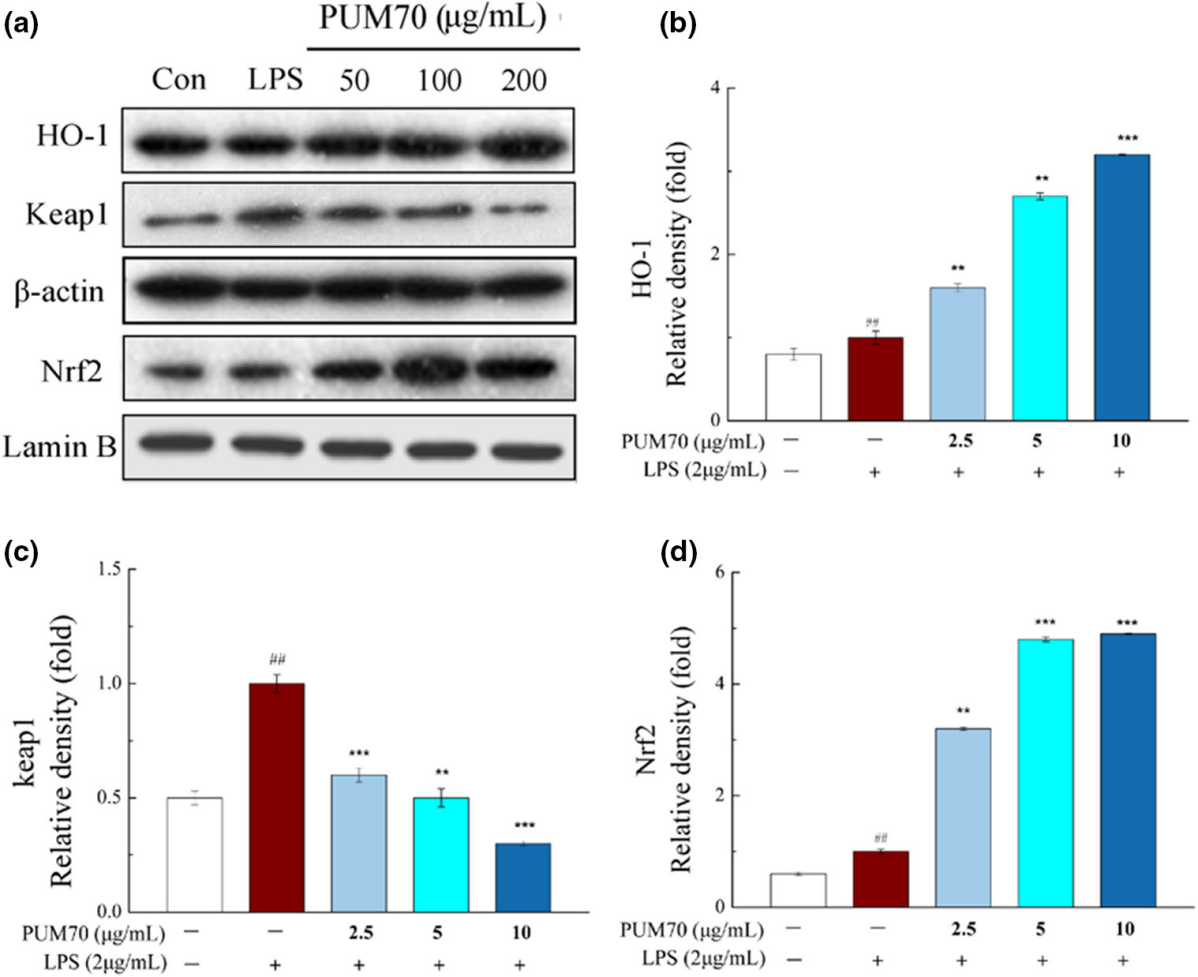
Effects of *Pyrus ussuriensis* Maxim 70% ethanol eluted fraction (PUM70) on HO‐1/Keap1/Nrf2 (heme oxygenase 1/Kelch‐like ECH‐associated protein 1/nuclear translocation of nuclear factor erythroid 2‐related factor 2) signaling pathway. (a) Expression of HO‐1, Nrf2, and Keap1; (b–d) the expression of iNOS, Nrf2, and Keap1. ^##^ *p* < .01 compared with control group; ***p < .001, **p < .01 compared with only lipopolysaccharide (LPS)‐treated cells

Nrf2/HO‐1 is an important endogenous antioxidant pathway. Here, western blot was performed to investigate the impact of PUM70 on the expression of HO‐1, Nrf2, and Keap1 (Kelch‐like ECH‐associated protein 1) induced by LPS (Figure 6a). The data demonstrated that PUM70 treatment significantly (*p* < .01) increased the intranuclear levels of HO‐1 and Nrf2 in the cells relative to the LPS group (Figure 6b,d). In addition, PUM70 treatment dramatically rescued the upregulation of cytosolic Keap1 caused by LPS stimulation (Figure 6c).

### 3.8 Effects of PUM70 on the phosphorylation levels of MAPKs

**FIGURE 7:**
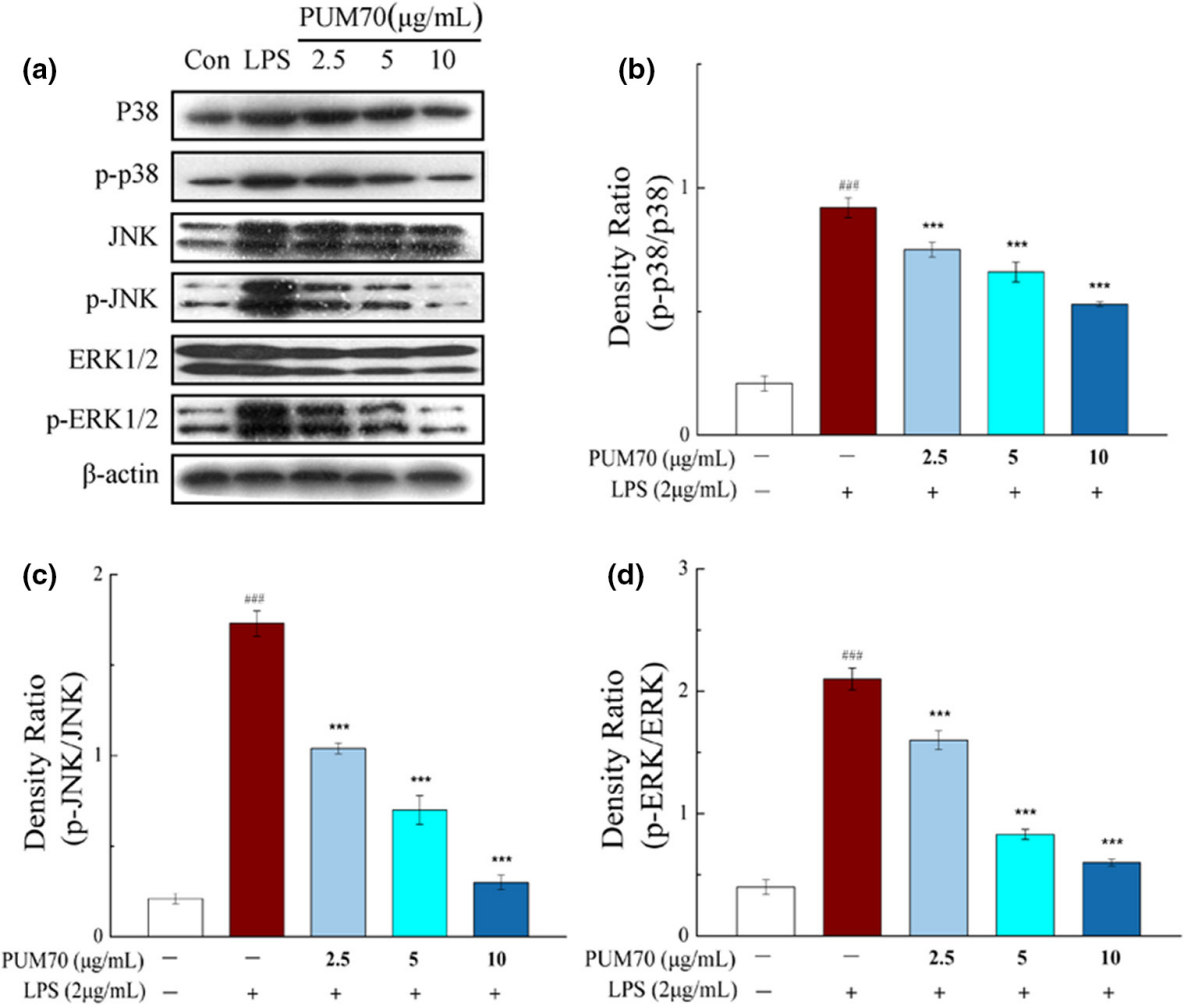
Effects of *Pyrus ussuriensis* Maxim 70% ethanol eluted fraction (PUM70) on mitogen‐activated protein kinase (MAPK) signaling pathway. (a) The phosphorylation of MAPKs; (b–d) the expression of phosphorylated p38, JNK (c‐Jun N‐terminal kinase), and ERK (extracellular signal‐regulated kinase). ^###^ *p* < .001 compared with control group; ***p < .001 compared with only lipopolysaccharide (LPS)‐treated cells

The results in Figure 7 indicate that there were significant increases in the phosphorylation of three MAPKs (p38, ERK (extracellular signal‐regulated kinase), and JNK (c‐Jun N‐terminal kinase)) in the LPS treatment group. However, the phosphorylation of MAPKs in the PUM70 treatment group was significantly inhibited, and the alleviating effect was correlated with the dose of PUM70 (Figure 7b–d).

## 4 DISCUSSION

Acute lung injury (ALI) is a common disease in clinical practice, whose specific molecular mechanism remains unclear due to its complexity and diversity. Changes in inflammatory response and vascular permeability generally occur at the initial stage of ALI (Zambelli et al., 2012). At the early stage of ALI, there will be an increase in the microcirculation permeability of lung tissue, leading to the extravasation of a large amount of plasma and proteins from blood vessels into the lung interstitium and alveolar cavity, and finally causing noncardiogenic pulmonary edema (Matthay et al., 2012). This is a pathologic feature of ALI and an important cause of progressive dyspnea and hypoxemia in patients. Therefore, a timely control of disease progression at the early stage of ALI is essential to the increase of cure rate. Recent studies have shown that some natural phytochemicals could inhibit inflammation by blocking the release of inflammatory cytokines and reduce the infiltration of pro‐inflammatory macrophages (Akanda et al., 2018; Raso et al., 2001). Numerous studies have revealed the biological effects of pear fruit, such as anti‐inflammatory and antioxidant effects, which can be mainly ascribed to the abundant phenolics in the fruit (Li et al., 2012). In this work, 18 phenolic compounds were identified in PUM70 using UPLC–MS/MS (ultraperformance liquid chromatography tandem mass spectroscopy), including flavan‐3‐ols, phenolic acids, anthocyanins, and flavonols. It has been reported that some phenolic compounds, such as kaempferol, quercetrin, and isoquercitrin, could prevent a variety of inflammatory diseases by activating the Nrf‐2 pathway (Comalada, et al., 2005; Li et al., 2016; Qian et al., 2019). Caffeic acids are known to have strong physiological activity, particularly in terms of anti‐inflammatory activity (Hong et al., 2015). As caffeic acid derivatives, caffeoylquinic acid activates Nrf2 and inhibits NF‐κB activation to prevent oxidative stress (Zhang et al., 2019). Therefore, the excellent antioxidant and anti‐inflammatory activity of PUM70 in this study may be closely related to these phenolic compounds.

It has been reported that ALI was associated with neutrophilic infiltration and increased inflammatory cytokines and exudation of proteins (Butt et al., 2016). At the early stage of ALI, neutrophils congregated at the site of lung injury to protect the host. However, excessive activation of neutrophils will cause damage to the tissue due to the release of various bioactive substances such as inflammatory cytokines and proteases (Grommes & Soehnlein, 2011). Numerous studies have shown that the decrease in neutrophils is related to the prognosis of ALI (Aulakh, 2018). Moreover, the release of pro‐inflammatory cytokines will activate neutrophils and further cause neutrophil infiltration, which may damage vascular epithelium and endothelial cells, followed by the occurrence of pulmonary edema due to the infiltration of plasma proteins into the interstitium of the lungs (Sharp et al., 2015). The excessive accumulation of neutrophils can also cause oxidative stress. ROS, as major oxidative components, is closely related to multiple signaling pathways triggered by inflammation‐related signal transduction cascades (Li & Engelhardt, 2006). It has also been revealed that ROS are potentially involved in the activation of nuclear factor‐kappa B (NF‐κB) signaling pathway at the early phase of several diseases (Nakajima & Kitamura, 2013). Thus, it is important to inhibit the overproduction of ROS for alleviating the oxidative stress caused by inflammatory cells. In addition, MDA could damage cell membranes, while antioxidant enzymes such as SOD and GSH can mitigate oxidative stress damage (Annapurna et al., 2013). This study confirmed that PUM70 can alleviate the pathological changes and oxidative stress injury of lung tissue.

The alveoli are the main sites of the body for gas exchange as well as the functional units of the lungs (Luo et al., 2018). Alveoli are mainly composed of small alveolar cells, large alveolar cells, and lung macrophages. Pneumonia occurs when pathogenic agents or pathogens enter and cause damage to the alveoli. Alveolar macrophages drive the inflammatory response in the lungs and are involved in many pulmonary inflammatory disorders (Wang et al., 2010). Therefore, the present study used mouse alveolar macrophages to investigate the effects of LPS stimulation on inflammatory pathways.

Mitogen‐activated protein kinase (MAPK) pathways are classic inflammatory signaling pathways, which can be activated by a variety of LPS‐induced inflammatory stimuli (Zhang et al., 2019). MAPKs belong to the serine/threonine protein kinase family, and include ERK, p38, and JNK as three major members. Phosphorylation of MAPKs due to various external stimuli activates the MAPK signaling pathway to promote the synthesis and secretion of iNOS, COX‐2, TNF‐α, IL‐1β, and IL‐6 (Gu et al., 2022; Kaminska, 2005). Our results clearly revealed that PUM70 inhibits the phosphorylation of three MAPKs in alveolar macrophages stimulated by LPS.

As another possible mechanism, LPS‐induced oxidative stress in ALI can be inhibited by the activation of Keap1/Nrf2/HO‐1 signaling pathway (Li et al., 2014). Nrf2 is normally sequestered by Keap1 in the cytosol. However, certain stimuli can trigger the release of Nrf2 from Keap1, facilitating the translocation of Nrf2 into the nucleus to activate HO‐1 (Balogun et al., 2003). HO‐1, an antioxidant enzyme, plays a critical role in preventing ROS damage to macrophages, and therefore has been intensively studied as a promising target in antioxidant studies (Araujo et al., 2012). Previous studies have shown that Nrf2 knockout mice are highly susceptible to oxygen‐induced lung injury and lung epithelial cell death (Cho et al., 2002; Papaiahgari et al., 2004). Under the nonactivated state, Nrf2 is confined to the cytoplasm. When the drug enters the cells, it will lead to a conformational change in Keap1, which loses its ability to block Nrf2 ubiquitination (Senthil et al., 2016). The undegraded Nrf2 is released and transferred to the nucleus, where it binds to the antioxidant response element (ARE), upregulates antioxidant enzymes and the HO‐1 protein, inhibits the production of ROS, and thus plays an antioxidant role (Taguchi et al., 2011). Therefore, we analyzed the protein level of Nrf2 in the nucleus to elucidate the mechanism for PUM70‐mediated Nrf2 activation. As a result, PUM70 significantly decreased the expression of Keap1 protein and increased those of Nrf2 and HO‐1 proteins. Similar to our results, some other plant‐derived compounds, such as resveratrol, curcumin, and sulfuretin, exert anti‐inflammatory effects via inducing HO‐1 expression (Chen et al., 2005; Jeong et al., 2009; Rushworth et al., 2006).

## 5 CONCLUSIONS

This study demonstrates that PUM70 which enriched phenolic compounds can ameliorate inflammation and oxidative stress in LPS‐induced ALI, resulting in an overall improvement of both macroscopic and histological parameters. The results also demonstrate that the protective effect of PUM70 on ALI damage caused by LPS may be related to the inhibition of MAPKs and Nrf2/HO‐1 signals. Further investigation may be focused on the protective effect of PUM70 using Nrf2‐knockout mice. The present study provides a foundation for the application of PUM70 as a potential anti‐inflammatory ingredient in functional foods or nutraceutical formulations.

## FUNDING INFORMATION

This research was funded by Hebei Province Science and Technology Support Program, grant number 20322803D.

## CONFLICT OF INTEREST

The authors declared no conflicts of interest.

## ETHICS STATEMENT

The animal study protocol was approved by the Ethical Committee for the Experimental use of animals at Hebei Normal University of Science and Technology (no. L2020109).

## Supporting information


**Figure S1** The total ion current chromatograms for *Pyrus ussuriensis* Maxim 70% ethanol eluted fraction (PUM70)

**Figure S2** The cell viability of alveolar macrophages

## Data Availability

The data that support the finding of this study are available from the corresponding author upon reasonable request.
